# Using a PyMOL Activity to Reinforce the Connection between Genotype and Phenotype in an Undergraduate Genetics Laboratory

**DOI:** 10.1371/journal.pone.0114257

**Published:** 2014-12-02

**Authors:** Alexandra D. Simmons, Thao K. T. Nguyen, Jack L. Follis, Albert Ribes-Zamora

**Affiliations:** 1 Department of Biology, University of Saint Thomas, 3800 Montrose Boulevard, Houston, Texas 770066–4626, United States of America; 2 Department of Mathematics, Computer Science and Cooperative Engineering, University of Saint Thomas, 3800 Montrose Boulevard, Houston, Texas 770066–4626, United States of America; University of Westminster, United Kingdom

## Abstract

With the purpose of developing an activity that would help clarify genetic concepts related to the connection between genotype and phenotype and the nature of mutations, we designed a three hour teaching module using the PyMol software. The activity starts with two pre-laboratory assignments, one to learn how to use PyMol and the other to read about a specific protein or protein family. During the laboratory students are given instructions where and how to find additional information on a specific disease and its causal mutations in order to prepare a 10-minute, in-class presentation. Using a post activity, anonymous quiz, we found a statistically significant different grade distribution in students that participated in the PyMol activity relative to a control group. We also found a significant improvement in the student’s comprehension when answering questions regarding the nature of mutations and protein structure. This demonstrates the utility of this simulation activity as a vehicle to improve student’s understanding of specific key genetic concepts.

## Introduction

For over a decade there has been a strong push from numerous recognized and well-accredited institutions and associations, such as the National Research Council, to change the way we teach science. Science teachers, professors and instructors have been compelled to enhance their teaching by using constructivist approaches, guided research, and student centered activities, which allow students to learn and explore science in a way that is more akin to the way scientists do science, and ultimately results in higher student engagement, retention and transfer of knowledge as compared to traditional lectures and laboratories [1], [2].

Computer simulations are defined as “computer-generated models that display theoretical or simplified models of real-world components, phenomena or processes” [3]. They allow interactive, authentic and meaningful learning opportunities to occur in situations where direct exploration would not be possible [3]. Research shows that using simulations can help develop content knowledge and process skills. Increased student understanding and achievement has been reported in physics, chemistry, biology and earth and space science [4]. Likewise, Rutten, van Joolingen, and van der Veena [5], agree that simulations have earned a place in the classroom as a robust addition that enriches teaching, either by complimenting or replacing traditional instruction, and that there are overall positive results when simulations are used to teach. The adaptive and interactive nature of computer software provides students a place to examine ideas or concepts through exploration, an environment in which to conduct experimentation or modeling that otherwise would not be possible in the classroom. Likewise, learning strategies can be individualized for each student, and one can include a variety of built-in assessment and activities that will allow learning to go beyond the classroom. In particular, simulations have been shown to increase conceptual change, skill development and content area knowledge, as it helps student visualize scientific concepts that are not observable in real life. Another advantage of IT-based teaching is that it can be structured around original sources of scientific data that span traditional disciplinary boundaries [1].

Genetics, as a subject, is difficult to teach because it involves mostly unobservable processes that occur at the cellular, molecular and organismal level, and many times requires quantitative analysis. Regarding genetics, there is evidence that shows that students exit high school with numerous misconceptions [6] and therefore enter their undergraduate genetics class with a collection of erroneous notions on how certain processes take place. One of the problems we have identified in our classrooms is that students have an over-simplistic notion of the connection between mutation and disease. There seems to be an incomplete understanding of the central dogma and therefore how a change in one or more nucleotides leads to a particular phenotype or phenotypes. In our experience, a high number of students fail to recognize how a particular phenotype, say for example a disease, can be caused by different types of mutations impacting multiple parts of a gene. In addition, they hold poor notions about the impact that said mutations can have on protein structure and function, which is a key concept for students to grasp in order to understand the genotype-phenotype connection, and moreover, the role that genetics and pharmacogenomics will play in the future of medicine.

With the aim of reinforcing the relationship between genotype and phenotype, we developed a 3-hour activity using PyMol, a protein visualization software (www.pymol.org), and other freely available genome browsers, in which teams of students learn about a specific genetic condition, explore a few specific causal mutations, and predict the potential impact those mutations may have on the overall structure and function of the protein. After the activity, we gave a short test to assess whether doing the PyMol activity had a positive impact on the student’s comprehension of this topic as compared to students who only learned the topic in lecture. We show data that supports that students that do the PyMol activity have a better understanding of the nature of mutations and general protein structure.

## Methods: Instructional Context and Implementation of the Activity

The University of St Thomas (UST) is a private, Catholic, Hispanic-serving, liberal arts institution, comprised of about 1600 undergraduate students. The Biology Department currently teaches undergraduate students only. Overall, we have approximately 265 students in the department and produce an average of 20 BA and BS graduates per year.

Genetics has been taught in the department since the program was established, but the Genetics Laboratory was only added in Spring 2012. Currently, the laboratory is mandatory for students pursuing a BS degree but not for those pursuing a BA. One of the general goals of the lab was to provide activities that would help clarify and reinforce genetic concepts that undergraduates find challenging. With this in mind we designed a 3-hour teaching module using the PyMol software, which can be downloaded for free for educational purposes, plus other genomic browsers. We do this exercise in the laboratory, but the activity can certainly be broken up into two or three parts to accommodate different schedules at other institutions.

Pre-lab handouts with background information on a specific protein or protein family are provided to the students one week before the day of the activity. These handouts are taken directly from the Molecule of the Month section of the Protein Data Bank webpage (http://pdb.org/pdb/101/motm_archive.do). Together with the handout, students are also given a homework assignment that allows them to begin exploring how to use the PyMol software (see File S1).

**Figure 1:**
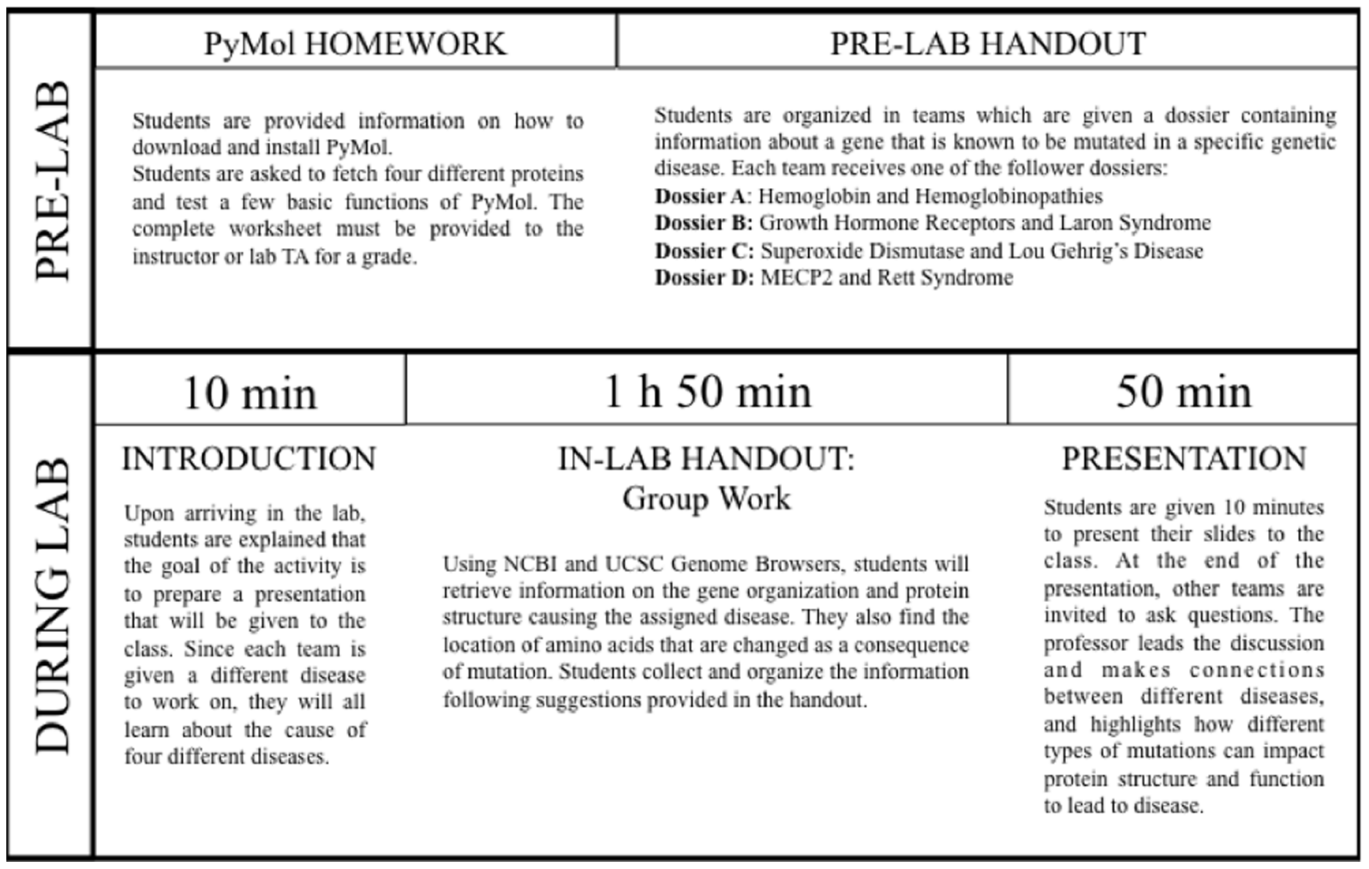
Overview of pre-lab and in-lab activities. For more details, please see File S1 and File S2.

On the day of the lab session, students hand in their homework and are given in-lab handouts that focus on a particular genetic disease. The handout contains a series of exercises that they must complete within two hours with the purpose of creating a 10 minutes presentation. The instructor is available to help students or troubleshoot when needed. In each laboratory section we have 16 students that are divided into teams of 4 individuals. Students are asked to bring at least 2 laptops per team (the department also owns some laptops that students can use during the activity). Each team receives a different in-lab handout that is related to the pre-lab handout they were given the previous week. We have developed a total of 4 handouts on the following topics: (i) Superoxide dismutase/Amyotrophic lateral sclerosis, (ii) Hemoglobin/Hemoglobinopathies, (iii) MECP2/Rett syndrome, and (iv) Growth hormone/Laron syndrome. Each handout contains the following sections: Short introduction, Gene organization, Protein structure, Disease associated mutations, and Presentation. The Introduction section briefly describes the activity and the protein students will be working with in lab. The Gene Organization section prompts students to find the location of the gene in the human genome, see how many introns and exons it has, and the number of amino acids each exon encodes for. In the Protein Structure section students are asked to investigate the function of their protein and its overall structure (number of alpha-helices, beta-sheets, quaternary structure). In the Disease-associated Mutation section, students are provided with a list of described amino acid changes that lead to the particular disease they are studying. These mutations have been selected to ensure that students are exposed to different types of changes, from simple missense mutations to early termination or frameshifts. They are asked to find the location of the mutation within the gene (ie, which exon), to predict how the change occurred at the nucleotide level, and the potential impact the change in amino acid would have on the overall protein shape and hence, its function. Last, the Presentation section gives some suggestions on how to organize their in-lab presentation (Figure 1). The pre-lab and in-lab handouts for the Hemoglobin/Hemoglobinopathies activity is included in File S2.

The teams are given two hours to complete the activity and during the last hour must give a 8–10 minute presentation using on their findings and show their models. Our teaching laboratories are outfitted with a projector and screen. Each team plugs in their laptop to the projector and can begin their short presentation. Most students use Office’s PowerPoint software (Microsoft) for their presentations. During each group’s talk, students are often asked to predict the effect of other hypothetical mutations on the protein structure and function and to reflect on whether a future therapeutic agent designed for one mutation would be effective in patients bearing other type of mutations.

## Results

After the activity we were interested in measuring if there was a better understanding of specific concepts among students that participated in the PyMol laboratory (PyMol group) as compared to students who were only enrolled in lecture and have never taken the laboratory (Control group). Two weeks after the lab session all three sections of Genetics lecture (66 students in total: 27 in the PyMol group and 39 in the Control group), were asked to complete an anonymous quiz that contained 22 questions that belonged to one of 9 concept categories related to the PyMol activity that are covered in the molecular portion of our genetic lecture (see Table 1). At UST, studies that focus on standard educational practices and/or tests do not require approval from the Human Subjects Committee. Nonetheless, students were asked to not identify themselves on the test and were informed that their quiz grade would not be included in their overall grade. They were also given the opportunity to opt out if they did not wish to participate and they were told that by taking the quiz they were consenting to participate in the study.

**Table 1:** Concept categories related to the PyMol activity that were assessed on the post-activity, anonymous quiz taken two weeks after completing the activity.

Concept/Topic	Quiz questions thataddressed the topic
DNA structure	1
Gene structure	3, 12
Protein structure	2, 7, 8, 14, 19, 20
Genetic code	4
Form fits function in proteins	9
Nature of mutations (change in DNA leads to changein protein sequence, which alters protein structure,and therefore function)	5, 6, 13, 15, 16, 17, 21
Mutation nomenclature	11
Change in one amino acid can alter protein structure,and therefore function	10, 22
Inheritability of mutations	18

On the quiz, the number of questions devoted to each concept was decided based on the need to have all concepts represented at least once on the quiz and the interest in a deeper assessment of those concepts more tightly linked to the PyMol activity. The inclusion of at least one question per concept also allowed us to asses the overall impact of this activity on the student’s comprehension of related genetic concepts, whereas the concentration of questions versing over protein structure and the nature of mutations provided the means to evaluate the effectiveness of this activity in reinforcing two concepts that constituted our specific student learning goals when we designed the activity.

**Figure 2:**
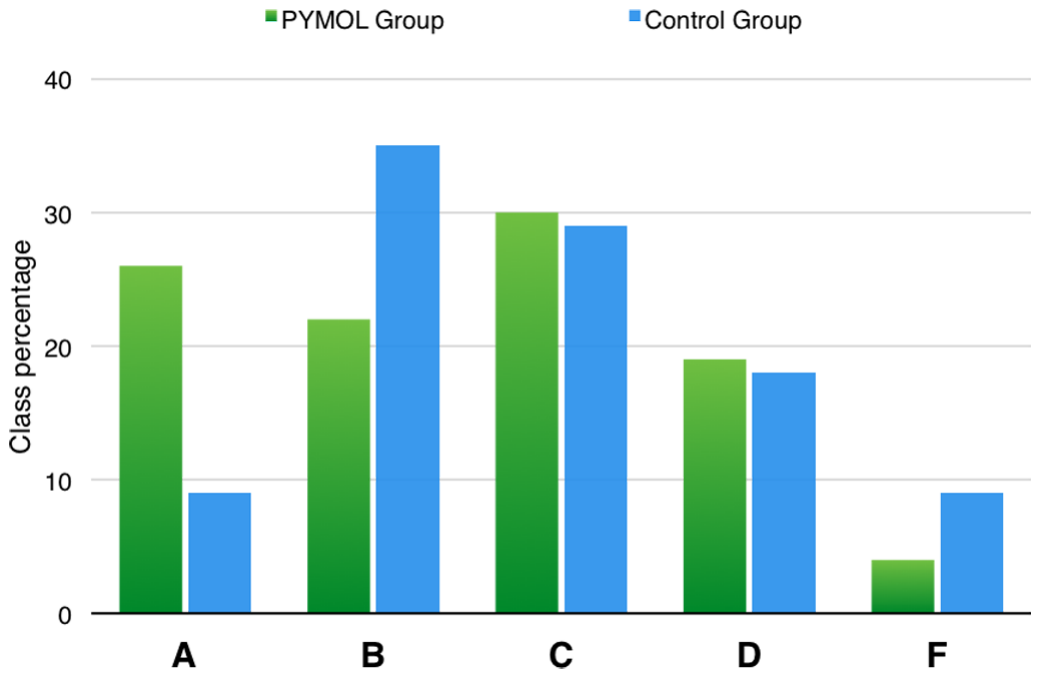
Letter grade distribution (percentage) of the overall grade made on the post-quiz. Grades were defined as follows: A = 90–100%, B = 80–89%, C = 70–79%, D = 60–69%, F = equal or below 59%.

When the average grade was calculated, we found that the average score for the PyMol group was 80.0 (SD = 10.9), while the average score for the Control group was 75.4 (SD = 14.4). We compared the averages using a t-test and obtained a p-value of 0.084. We then examined the grade distribution in each group. Figure 2 shows the grade distribution observed in the PyMol and Control groups when all questions are considered. To test for differences in the distribution of grades between the two groups, a chi-squared goodness of fit test was used. The distribution of grades for the control group was used to calculate the expected values. While grade distribution for the control group is close to a normal distribution, students that participated in the PyMol lab show a statistically significant distribution shift (p = 0.027) towards higher letter grade. This shows that the PyMol activity improved student’s overall comprehension of key concepts in molecular genetics related to the phenotype-genotype connection.

**Figure 3:**
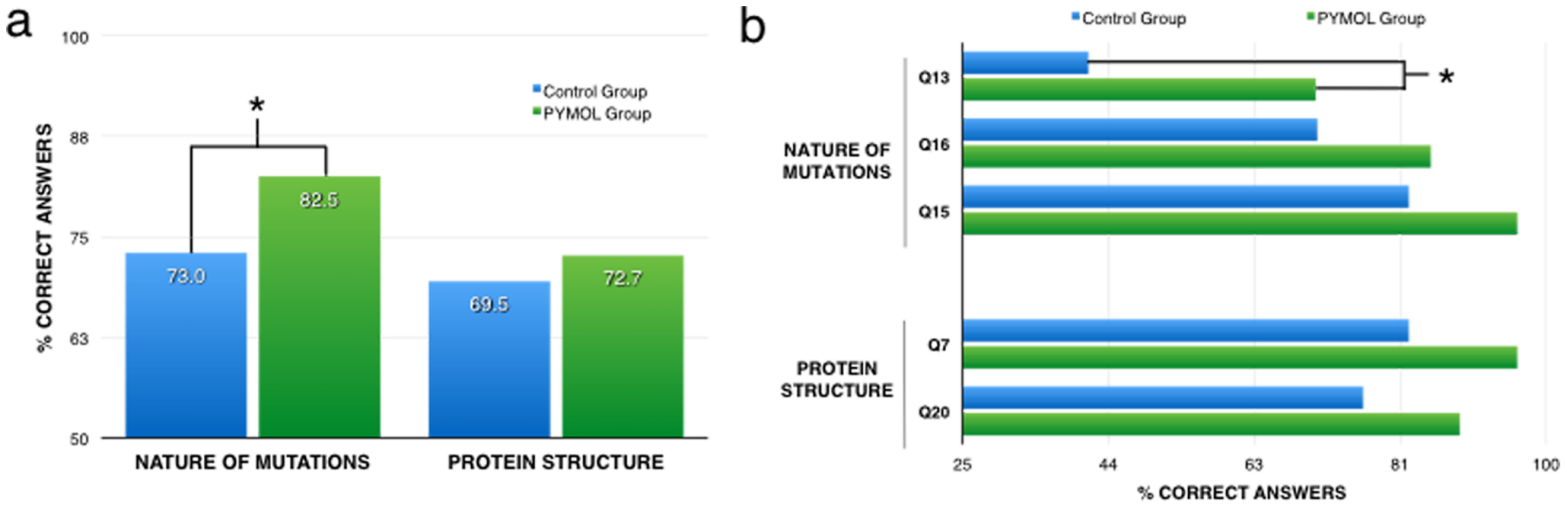
Effects of PyMol activity on student comprehension regarding the nature of mutations and protein structure. (a) Percentage of correct responses of the PyMol and Control groups for those questions that assess either student’s comprehension of the nature of mutations or protein structure. Each bar represents the collective data obtained when combining the responses of all questions pertaining to either category. (b) Questions belonging to the two genetic concepts analyzed in A that display at least a 10 point difference in the percentage of correct answers between PyMol and Control groups. Asterisks represent p<0.05.

Next, we shifted our attention towards those concepts that were the major focus of the Pymol activity: nature of mutations and protein structure. Using a t-test, we first compared student’s performance between PyMol and Control groups when protein structure and nature of mutation questions were aggregated. We found that the average score for the PyMol group was 77.78 (SD = 11.15), whereas the average score of the Control group was 71.72 (SD = 15.47). These averages are statistically significant with a p value of 0.04. Next we analyzed these two topics separately. As shown in Figure 3a, we found a statistically significant increase (p = 0.02) of nearly 10 percentage points in the students ability to correctly respond to questions about the nature of mutations if they participated in the PyMol lab. Similarly, after doing the Pymol activity, students performed better than control group when confronted with questions dealing with protein structure, although in this case the difference was not statistically significant (p = 0.24).

To test for an association between lab activity and the proportion of correct answers for individual questions, the Pearson chi-squared test was used when there were at least 5 correct and 5 incorrect answers for each group. For questions that had less than 5 correct or incorrect answers for either group, Barnard’s test was used. All tests were conducted using R version 3.0.2 [7], including the package ‘exact’ for Barnard’s test. When looking at individual questions belonging to either the nature of mutations or the protein structure categories, we found a total of 7 questions were the difference between the two groups was above 10 percentage points (Figure 3b). In the case of Q13, the difference between both groups was big enough to be considered statistically significant (p = 0.023). This question asks students to identify whether or not disease related mutations can be found in gene parts other that exons. Q16 asks if mutations in the coding region always lead to a change in the protein, whereas Q15 asks them whether it is true or not that all mutations will affect the structure of proteins. With respect to protein structure specific questions, Q7 asks students whether it is true or false that all proteins are made of a single peptide and Q20 inquires about the nature of amino acids found in the core of globular proteins.

Overall this data indicates that the Pymol activity can improve students’ comprehension of key molecular genetics concepts and, in particular, concepts that clarify the complex mechanism by which mutations can disable proteins or disrupt their function, and hence how the flow of genetic information occurs from gene to protein, as reflected in the central dogma. This provides a strong foundation that we expect will facilitate their comprehension of concepts explored in upper division classes (Molecular Biology, Cell Biology, Cancer), such as those related to the new fields of personalized medicine and pharmacogenomics.

## Discussion and Conclusions

With the purpose of helping our students overcome some basic misconceptions in the area of genetics and providing a more effective tool to help them develop a more sophisticated understanding of the connection between genotype and phenotype, we designed a 3-hour activity using PyMol software in which students explore how mutations can lead to proteins that may be truncated or have impaired function, and therefore, cause specific diseases. By means of a 22 question post-quiz we assessed whether students enrolled in lecture that participated in the PyMol activity in lab (PyMol group) performed better than students that learned the same concepts in lecture only (Control group). In general terms, we found that the PyMol group achieved better results than the Control group.

We are aware that our study has some limitations. Because of the characteristics of our university, class size is typically small. These results in a loss of power for our study due to the small sample size (n = 61). While we can see meaningful differences between the groups, we are limited in our ability to detect statistically significant results. But we have found that this should not be discouraging to other groups interested in developing new teaching strategies and conducing science education research in small classes, as even in these conditions one can detect some significant differences in student performance. Another caveat of the present study is that our test group is smaller than our control group, but again, here we are limited to the number of students that enroll in the laboratory, which is capped at 16 seats, and that only BS students are required to take the laboratory. One more thing that we believe must be taken into account is that it is entirely possible that BS students may be more motivated or stronger students that those pursuing a BA. From Spring 2012 to Spring 2014 the average GPA of students completing a Biology BS degree was 3.558, whereas the average GPA of students completing a Biology BA degree was 3.400. The GPAs seem to fluctuate a bit from year to year, but the difference seems to support the idea that BS students may be slightly stronger or more highly motivated. However, this analysis goes beyond the scope of this paper and should be addressed in a separate study. We also noticed that using some of the browsers (NCBI’s Map Viewer, UCSC’s Genome Browser) could be somewhat intimidating to students. In these instances is when we reaffirmed the value of having the instructor in the lab with the students, as we were instrumental in helping our students navigate through some of the less intuitive websites, and ultimately, make the necessary connections to learn.

Nonetheless, our overall experience was extremely positive. We saw a significant shift in grade distribution (towards higher grades) when comparing the PyMol and Control groups. We showed that the general and activity-specific questions were answered correctly more often in our PyMol group than in our Control group. Consequently, we have shown that using the PyMol activity has allowed our students to clarify the mechanism by which mutations can lead to disease in the context of the central dogma; that is, that a change in the DNA molecule may change an amino acid to a stop codon and lead to the formation of a truncated protein, that the change at the DNA level may lead to the insertion of a different amino acid that may alter the shape of the protein or negatively impact protein-protein interaction that may be relevant to the protein’s overall function, or that the change in the DNA molecule may lead to a splice variant that likewise produces a physically altered, non-functional protein. It is important to note that the PyMol software itself does not allow amino acid modification: students can only highlight specific amino acids to pinpoint their location in the 3D structure of the protein. However, the activity is conducive to discussions about the potential impact of these modifications. Students know that all the mutations we ask them to explore are causal mutations, and therefore they are invited to predict the consequence of the alteration. They become involved with the subject, build hypotheses and carry out explanations using the correct technical vocabulary. We believe that the discussion during and after the presentations is equally important as doing the activity, as here is where the real learning takes place. In a similar study, Craciun and Isvoran [8] used programs available online (PDB, SWISS PROT, PyMol, First Glance, RasMol, Kinemage, BLAST, CLUSTALW, T-Coffee, SOSUI, PCE, Qgrid, SCREEN, etc) to study the physicochemical properties and prediction of structures and functions of biological molecules for the course “Molecular basis of therapeutics action”. Likewise, they report numerous advantages of using Internet tools of molecular visualization, such as positive student feedback, using in the classroom software that is currently being used in research, and the fact that these type of activities allow for a student-centered learning environment.

Last, even though we did not use a specific tool to assess student engagement, we can attest that students were highly engaged during the PyMol activity. We observed that each team member seemed to have a set of designated tasks to contribute to the overall project, but all members share and help each other. This lab was loud, as we witnessed a lot of dynamic communication happening within teams and even between teams. In an end of semester survey, students were asked to chose their top 3 favorite activities done in the lab: PyMol was the third most popular selection after learning specific lab techniques in the context of a research project and conducting an guided research project in the lab.

## Supporting Information

File S1 **PyMol pre-lab homework.**
(PDF)

File S2 **Example of in-class handout.**
(PDF)
