# Supplementary material for: Using a PyMOL Activity to Reinforce the Connection between Genotype and Phenotype in an Undergraduate Genetics Laboratory
Source: PLoS One. 2014 Dec 2;9(12):e114257. doi: 10.1371/journal.pone.0114257 (PMC4252116; doi:10.1371/journal.pone.0114257)
Supplement: File S1 — PyMol pre-lab homework. (PDF) [file pone.0114257.s001.pdf]

PyMol Homework

NAME:

In LAB 8 we will explore Gene and Protein Structure. We will be using Pymol throughout the lab and thus, it is important that you come to class having practiced how to work with this software. You instructor will give you a short demo during a previous lab or in lecture. There are also many PyMol tutorials available online. For a basic tutorial, check out this YouTube video: http://youtu.be/RAftPWs1sWQ.

Go to the PyMol website and download the Educational-use-only version of the software (http://pymol.org/educational/). Follow the website's instructions for installation.

USE WHITE BACKGROUND (Click Display > Background > White).

1. **GROEL chaperon** (PDB: **1AON)**

This is a big complex that helps proteins fold properly.

1. Open the structure (type 'fetch 1aon' in the command line window or download the structure from pdb.org).

2. Show each chain with a different color (in right hand panel, click 'all > C > by chain > by chain').

3. Depict structure as 'Surface' (all > S > surface).

Once you are done select a nice angle, ray the image it if possible (button on top right panel), make the background white (display > background > white), save the image and paste it in the box on the right.

2. **Nucleosome** (PDB: **1AOI)**

If you click "Display > sequence on", you can see the full sequence for both DNA and protein. This also allows you to manually select certain sections and manipulate them.

Depict histones as 'surface' and show DNA as 'cartoon'.

Select: (1aoi) → 'A' → 'generate'→ 'vaccum electrostatics' → 'protein contact potential'

In this figure, blue regions represent basic areas whereas red regions represent acidic areas. Why do you think they are so many positive residues in the areas that contact DNA? (To show DNA again, click on 1aoi)

Once you are done select a nice view, ray it if possible, save the image and past it in the box to the right.

3. **TBP: TATA-Box Binding Protein** (PDB: **1TGH**)

This is the protein that binds TATA-BOX sequence in promoters and initiates assembly of the transcriptome.

1. Load the file, hide the water molecules, and show protein as 'cartoon' only (you need to hide 'lines'). Show the DNA as 'sticks'.

2. Color protein in your favorite shade of blue.

3. Select L281, color it red and show that residue as 'spheres'

4. Select K191, color it red and show that residue as 'spheres'

Select a nice view of the molecule, ray it if possible and paste the image in the box on the right.

4. **RIBOSOME** (PDBs: **2WDK & 2WDL**)

1.Fetch 2wdk (small subunit). From the top of the screen, click Display > Sequence mode > Chains. Numbers and letters will appear on the top of the screen. As you can see chain A and V through Y correspond to r-RNA whereas the rest of chains (B through U) correspond to protein subunits. You can also figure this out by displaying residues instead of chains and looking at the composition of each subunit: RNA chains will be made of A, C, G and U, whereas protein subunits will be represented as a sequence of amino acids.

2. Color all r-RNA in the small subunit in your favorite shade of green. Show them as 'cartoon'.

3. Select all proteins in the small subunit and show them as 'spheres'.

4.Fetch 2wdl (large subunit)

5. Color the large subunit using your favorite shade of blue.

6. Select all protein chains in the large subunit (1 through 9 and C through Z) and show them as 'spheres'.

7. Select all r-RNA chains in the large subunit (A and B) and show them as 'cartoon'.

Select a nice view, ray it if possible and paste the image in the box on the right.
