# Supplementary material for: Using a PyMOL Activity to Reinforce the Connection between Genotype and Phenotype in an Undergraduate Genetics Laboratory
Source: PLoS One. 2014 Dec 2;9(12):e114257. doi: 10.1371/journal.pone.0114257 (PMC4252116; doi:10.1371/journal.pone.0114257)
Supplement: File S2 — Example of in-class handout. (PDF) [file pone.0114257.s002.pdf]

HEMOGLOBINOPATHIES

Today your job will be to study a particular gene from its organization and location in the genome to the structure of the protein it encodes for. You have to gather information, interpret it and present your findings in a formal 8 minutes class talk.

You have been assigned to study Hemoglobin which is encoded by two similar genes: HBA (alpha-hemoglobin) and HBB (Beta-hemoglobin). These two genes are important gene because of their physiological role and because they have been found mutated in patients suffering from several diseases including sickle cell anemia and B-thalassemia. Before you start, make sure you read the introductory information that accompanies this dossier.

Here the elements that needs to appear in your presentation

GENE ORGANIZATION

- Use the three websites listed below to find where these genes are located in the genome.
- How many introns and exons does each gene have?
- How many amino acids does each exon encode?

  Note on exon information: You can find additional information on the amino acids that make up each exon in a gene by going to UCSC and clicking on the gene of interest. Click on 'Protein FASTA' (located in the second table) and in Formatting Options check 'separate exons'. Click 'submit'. You will obtain a readout that looks like this:

  ```
  >uc002tfm.4_hg19_1_20 23 0 0 chr2:110962477-110962545-
  MLARRQRDPLQALRRRNQELKQQ
  ```

  hg19 is the Human Genome Assembly Number (version). 1_20 represents that the gene has 20 exons and you are looking at the first one. The number '23' indicates that there are 23 amino acids in that particular exon. Below is the amino acid sequence.

We recommend that you make a graphical representation of your genes where we can see its organization in the genome and show the mRNAs it can produce. Here is an example:

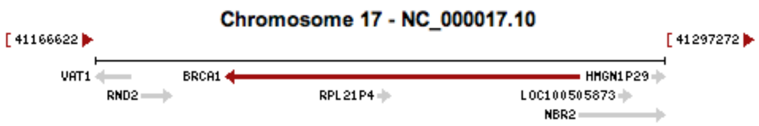

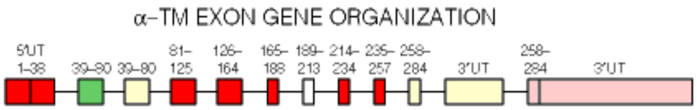

In order to answer the previous questions, please use the following links:

Map viewer:

HBA (http://www.ncbi.nlm.nih.gov/nuccore/NC_000016.9?from=226552&to=227645&report=graph&content=5)

HBB: (http://www.ncbi.nlm.nih.gov/nuccore/NG_000007.3?from=70303&to=72390&report=graph&content=5)

UCSC Genome Browser:

HBA (http://genome.ucsc.edu/cgi-bin/hgTracks?position=chr16:226679-227520&hgsid=294828783&refGene=pack&hgFind.matches=NM_000558,)

HBB (http://genome.ucsc.edu/cgi-bin/hgTracks?position=chr11:5246696-5248301&hgsid=294828783&knownGene=pack&hgFind.matches=uc001mae.1,)

NCBI gene:

HBA (http://www.ncbi.nlm.nih.gov/gene/3039)

HBB (http://www.ncbi.nlm.nih.gov/gene/3043)

PROTEIN STRUCTURE

- What is the physiological role of hemoglobin?
- What is the function of the Heme group? In the protein sequence, the heme group will be abbreviated as HEM.

We strongly recommend that you use different views of the hemoglobin pymol structure to make your points during your presentation. Make sure that you mark the Heme group with another color. Here is the PDB identifier for hemoglobin: 2hhb

DISEASE-ASSOCIATED MUTATIONS

Below you will find a list of human mutations found in hemoglobin.
HBA: K17V, D76A and D127Y.
HBB: E7V, H93N and H144D.

- *Note on mutation abbreviations: Take K17V for example. This abbreviation means that normal amino acid K (lysine) in position 17 has been substituted my amino acid V (valine) in the individual with the mutation.*
- *To further help you out, your instructor will provide you with an additional sheet with the cDNA and amino acid sequence of your gene to help you with the following exercise.*

1. First, locate each mutation in the gene organization figure that you produced earlier. It should look similar to the example below.

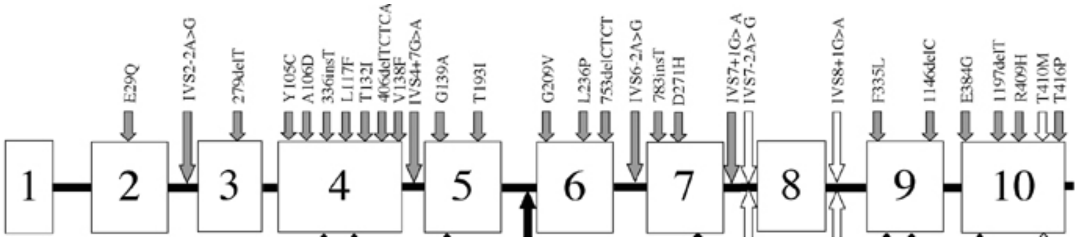

2. Scientists have experimentally found that mutation H93N in HBB makes the protein completely unstable. *Notice the following: the pymol structure is missing the first amino acid so everything is shifted one position, so position #93 actually appears in #92.*

   a. The codon that encodes for histidine at amino acid #93 is CAC. Predict what type of changes, AT THE NUCLEOTIDE LEVEL, may have produced a change from histidine to asparagine. Use the genetic code provided in this dossier.
   b. Produce a pymol figure clearly highlighting position 93 so that you can explain during your presentation why you think that a change in that position may cause protein instability.

3. Scientists have also found that mutation E7V in HBB that causes hemoglobin to form long fibrils that induces a disease called sickle cell anemia.

   a. Indentify the codon that encodes for glutamic acid at amino acid #7 and predict what type of changes, AT THE NUCLEOTIDE LEVEL, may have produced a change from glutamic acid to valine. Use the genetic code provided in this dossier.
   b. Open E7V mutant pymol structure 2hbs and produce a pymol figure showing the location of the mutation. During your presentation, use this figure to explain how this mutation can cause sickle cell anemia.

4. Scientist have also found that the codon that encodes for a lysine in position #18 (HBB) can be found changed from AAG to TAG in patients with beta-thalassemia, a hemoglobinopathy where beta-hemoglobin is produced in lower amounts than alpha-hemoglobin. Explain in your presentation why do you think this mutation may cause this disease.

Presentation

You are free to add more interesting aspects about your genes if you have time, but make sure that all elements from above show up in your presentation.

Here is a suggestion about how to structure your talk:

1- Introduction to Hemoglobin and its physiological role.
2- Structure of alpha-Hemoglobin and beta-Hemoglobin genes.
3- Structure of hemoglobulin (or hemoglobin).
4- Disease related mutations.
5- Mutation H93N.
6- Mutation E7V and sickle cell anemia.
7- Beta-thalassemia producing mutation.

When presenting your mutations consider the IMPACT of the amino acid change. Does it change the charge? Does it change the shape or add bulk? Look at the amino acid table properties to guide you through your thought process.

Make sure you practice what you will say in each slide.

GENETIC CODE

First Position		Second Position of Codon: T	C	A	G		Third Position
	T	TTT Phe [F]	TCT Ser [S]	TAT Tyr [Y]	TGT Cys [C]	T	
		TTC Phe [F]	TCC Ser [S]	TAC Tyr [Y]	TGC Cys [C]	C	
		TTA Leu [L]	TCA Ser [S]	TAA *Ter* [end]	TGA *Ter* [end]	A	
		TTG Leu [L]	TCG Ser [S]	TAG *Ter* [end]	TGG Trp [W]	G	
	C	CTT Leu [L]	CCT Pro [P]	CAT His [H]	CGT Arg [R]	T	
		CTC Leu [L]	CCC Pro [P]	CAC His [H]	CGC Arg [R]	C	
		CTA Leu [L]	CCA Pro [P]	CAA Gln [Q]	CGA Arg [R]	A	
		CTG Leu [L]	CCG Pro [P]	CAG Gln [Q]	CGG Arg [R]	G	
	A	ATT Ile [I]	ACT Thr [T]	AAT Asn [N]	AGT Ser [S]	T	
		ATC Ile [I]	ACC Thr [T]	AAC Asn [N]	AGC Ser [S]	C	
		ATA Ile [I]	ACA Thr [T]	AAA Lys [K]	AGA Arg [R]	A	
		ATG Met [M]	ACG Thr [T]	AAG Lys [K]	AGG Arg [R]	G	
	G	GTT Val [V]	GCT Ala [A]	GAT Asp [D]	GGT Gly [G]	T	
		GTC Val [V]	GCC Ala [A]	GAC Asp [D]	GGC Gly [G]	C	
		GTA Val [V]	GCA Ala [A]	GAA Glu [E]	GGA Gly [G]	A	
		GTG Val [V]	GCG Ala [A]	GAG Glu [E]	GGG Gly [G]	G	

Amino Acid Properties Table

Small

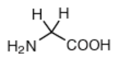

Glycine (Gly, G)
MW: 57.05

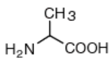

Alanine (Ala, A)
MW: 71.09

Nucleophilic

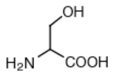

Serine (Ser, S)
MW: 87.08, pK_a ~ 16

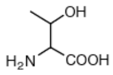

Threonine (Thr, T)
MW: 101.11, pK_a ~ 16

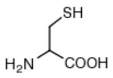

Cysteine (Cys, C)
MW: 103.15, pK_a = 8.35

Hydrophobic

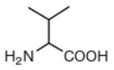

Valine (Val, V)
MW: 99.14

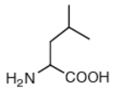

Leucine (Leu, L)
MW: 113.16

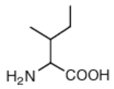

Isoleucine (Ile, I)
MW: 113.16

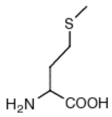

Methionine (Met, M)
MW: 131.19

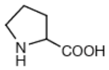

Proline (Pro, P)
MW: 97.12

Aromatic

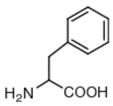

Phenylalanine (Phe, F)
MW: 147.18

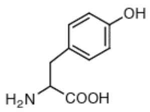

Tyrosine (Tyr, Y)
MW: 163.18

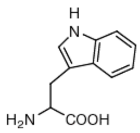

Tryptophan (Trp, W)
MW: 186.21

Acidic

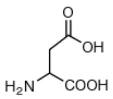

Aspartic Acid (Asp, D)
MW: 115.09, pK_a = 3.9

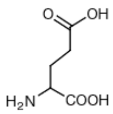

Glutamic Acid (Glu, E)
MW: 129.12, pK_a = 4.07

Amide

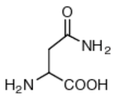

Asparagine (Asn, N)
MW: 114.11

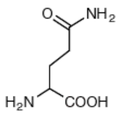

Glutamine (Gln, Q)
MW: 128.14

Basic

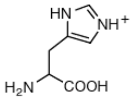

Histidine (His, H)
MW: 137.14, pK_a = 6.04

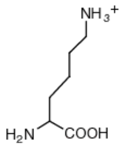

Lysine (Lys, K)
MW: 128.17, pK_a = 10.79

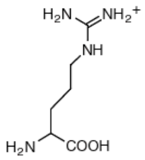

Arginine (Arg, R)
MW: 156.19, pK_a = 12.48

HBA1


```
  1  ATGGTGCTGTCTCCTGCCGACAAGACCAACGTCAAGGCCGCCTGGGGTAAGGTCGGCGCGCACGCTGGCGAGTATGGTGCGGAGGCCCTGGAGAGGATGT
  1▶ M  V  L  S  P  A  D  K  T  N  V  K  A  A  W  G  K  V  G  A  H  A  G  E  Y  G  A  E  A  L  E  R  M
101  TCCTGTCCTTCCCCACCACCAAGACCTACTTCCCGCACTTCGACCTGAGCCACGGCTCTGCCCAGGTTAAGGGCCACGGCAAGAAGGTGGCCGACGCGCT
 34▶F  L  S  F  P  T  T  K  T  Y  F  P  H  F  D  L  S  H  G  S  A  Q  V  K  G  H  G  K  K  V  A  D  A  L
201  GACCAACGCCGTGGCGCACGTGGACGACATGCCCAACGCGCTGTCCGCCCTGAGCGACCTGCACGCGCACAAGCTTCGGGTGGACCCGGTCAACTTCAAG
 67▶  T  N  A  V  A  H  V  D  D  M  P  N  A  L  S  A  L  S  D  L  H  A  H  K  L  R  V  D  P  V  N  F  K
301  CTCCTAAGCCACTGCCTGCTGGTGACCCTGGCCGCCCACCTCCCCGCCGAGTTCACCCCTGCGGTGCACGCCTCCCTGGACAAGTTCCTGGCTTCTGTGA
101▶ L  L  S  H  C  L  L  V  T  L  A  A  H  L  P  A  E  F  T  P  A  V  H  A  S  L  D  K  F  L  A  S  V
401  GCACCGTGCTGACCTCCAAATACCGTTAA
134▶S  T  V  L  T  S  K  Y  R
```

HBB


```
  1  ATGGTGCATCTGACTCCTGAGGAGAAGTCTGCCGTTACTGCCCTGTGGGGCAAGGTGAACGTGGATGAAGTTGGTGGTGAGGCCCTGGGCAGGCTGCTGG
  1▶ M  V  H  L  T  P  E  E  K  S  A  V  T  A  L  W  G  K  V  N  V  D  E  V  G  G  E  A  L  G  R  L  L
101  TGGTCTACCCTTGGACCCAGAGGTTCTTTGAGTCCTTTGGGGATCTGTCCACTCCTGATGCTGTTATGGGCAACCCTAAGGTGAAGGCTCATGGCAAGAA
 34▶V  V  Y  P  W  T  Q  R  F  F  E  S  F  G  D  L  S  T  P  D  A  V  M  G  N  P  K  V  K  A  H  G  K  K
201  AGTGCTCGGTGCCTTTAGTGATGGCCTGGCTCACCTGGACAACCTCAAGGGCACCTTTGCCACACTGAGTGAGCTGCACTGTGACAAGCTGCACGTGGAT
 67▶  V  L  G  A  F  S  D  G  L  A  H  L  D  N  L  K  G  T  F  A  T  L  S  E  L  H  C  D  K  L  H  V  D
301  CCTGAGAACTTCAGGCTCCTGGGCAACGTGCTGGTCTGTGTGCTGGCCCATCACTTTGGCAAAGAATTCACCCCACCAGTGCAGGCTGCCTATCAGAAAG
101▶ P  E  N  F  R  L  L  G  N  V  L  V  C  V  L  A  H  H  F  G  K  E  F  T  P  P  V  Q  A  A  Y  Q  K
401  TGGTGGCTGGTGTGGCTAATGCCCTGGCCCACAAGTATCACTAA
134▶V  V  A  G  V  A  N  A  L  A  H  K  Y  H
```
